# State-of-the-art review article. Atherosclerosis affecting fat: What can we learn by imaging perivascular adipose tissue?

**DOI:** 10.1016/j.jcct.2019.03.006

**Published:** 2019

**Authors:** Charalambos Antoniades, Christos P. Kotanidis, Daniel S. Berman

**Affiliations:** a Division of Cardiovascular Medicine, Radcliffe Department of Medicine, University of Oxford, Oxford, UK; b Department of Imaging and Medicine, Cedars-Sinai Medical Center, Los Angeles, CA, USA

**Keywords:** Perivascular adipose tissue, Atherosclerosis, Inflammation, Computed tomography imaging

## Abstract

Perivascular adipose tissue (PVAT) surrounding the human coronary arteries, secretes a wide range of adipocytokines affecting the biology of the adjacent vascular wall in a paracrine way. However, we have recently found that PVAT also behaves as a sensor of signals coming from the vascular wall, to which it reacts by changing its morphology and secretory profile. Indeed, vascular inflammation, a key feature of vascular disease pathogenesis, leads to the release of inflammatory signals that disseminate into local fat, inducing local lipolysis and inhibiting adipogenesis. This ability of PVAT to sense inflammatory signals from the vascular wall, can be used as a “thermometer” of the vascular wall, allowing for non-invasive detection of coronary inflammation. Vascular inflammation induces a shift of PVAT's composition from lipid to aqueous phase, resulting into increased computed tomography (CT) attenuation around the inflamed artery, forming a gradient with increasing attenuation closer to the inflamed coronary artery wall. These spatial changes in PVAT's attenuation are easily detected around culprit lesions during acute coronary syndromes. A new biomarker designed to captured these spatial changes in PVAT's attenuation around the human coronary arteries, the Fat Attenuation Index (FAI), has additional predictive value in stable patients for cardiac mortality and non-fatal heart attacks, above the prediction provided by the current state of the art that includes risk factors, calcium score and presence of high risk plaque features. The use of perivascular FAI in clinical practice may change the way we interpret cardiovascular CT angiography, as it is applicable to any coronary CT angiogram, and it offers dynamic information about the inflammatory burden of the coronary arteries, providing potential guidance for preventive measures and invasive treatments.

## Abbreviations

AT: Adipose Tissue
EAT: Epicardial AT
SAT: Subcutaneous AT
VAT: Visceral AT
PVAT: Perivascular AT
NO: Nitric oxide
eNOS: Endothelial NO Synthase
CTA: Computed Tomography Angiography
SPECT: Single-Photon Emission Computed Tomography
PET: Positron Emission Tomography
FAI: Fat Attenuation Index
TNFα: Tumor Necrosis Factor alpha
IL-6: Interleukin 6
IFNγ: Interferon gamma

## 1 Introduction

Ischemic heart disease remains the leading cause of death in upper-middle and high income economies.^1^ Coronary artery disease (CAD) accounts for 1 in 7 deaths in the United States, responsible for over 366,800 deaths every year, with associated healthcare costs continuing to rise and projected to reach $749 billion by 2035.^2^ Despite progress in primary and secondary prevention, significant residual cardiovascular risk persists.^3^ Although novel and effective (but also expensive) therapies target the residual cardiovascular risk (e.g. anti-PCSK9 monoclonal antibodies^4^ or canakinumab^5^), their clinical adoption by healthcare systems remains low. It is therefore time to consider developing “companion diagnostics”, i.e. tests that will allow us to tailor deployment of these new and expensive therapeutics in well-defined populations who can derive maximum benefit, entering the era of personalized (or precision) medicine. New diagnostic tests allowing detection of the “vulnerable patient” will enable application of targeted treatments in primary or secondary prevention, to prevent the development of clinical cardiovascular disease, including myocardial infarction. Further, these tests might allow identification of patients with severe but stable CAD in whom unnecessary invasive therapy might be avoided. However, understanding the potential implications of these new diagnostic tests requires knowledge of the factors behind “residual cardiovascular risk”, not currently captured by the clinical or biochemical risk scores.

### 1.1 Atherogenesis and inflammation

Inflammation has long been considered to be a central driver of atherogenesis, as well as being a key element in the development of the vulnerable atherosclerotic plaque.^6^ Both arms of immunity, innate and adaptive, are implicated in a complex network of molecular and cellular interactions that comprise the inflammatory reaction at the atherosclerotic site.^7^^,^^8^ Systemic markers of inflammation, mainly high sensitivity C-reactive protein (hsCRP) and proinflammatory cytokines, have been associated with cardiovascular risk prediction, independently of established cardiovascular risk factors, and the concept of residual inflammatory risk has been proposed.^9^ This hypothesis was strengthened by clinical trials like JUPITER,^10^ in which rosuvastatin reduced cardiovascular risk in individuals with elevated hsCRP. However, the risk reduction was in line with that expected by LDL lowering alone, so the contribution of rosuvastatin's pleiotropic anti-inflammatory effects was not fully documented. It was only in 2017 that the CANTOS trial^5^ documented for the first time in humans that targeting IL1β using canakinumab reduced cardiovascular events, proving the causal role of inflammation in human disease pathogenesis. Despite the well-established role of inflammation in vascular disease pathogenesis, it is still unclear how to select patients with high levels of vascular inflammation, who would benefit most by targeting inflammation.^11^ Circulating biomarkers like hsCRP or IL6, are excellent in detecting systemic inflammation that usually co-exists with coronary inflammation, but they are often driven by other systemic or local inflammatory conditions like arthritis, infections and other conditions.^12^ Therefore, understanding the role of inflammation in atherogenesis, in particular the local inflammation-induced changes in the human coronary arteries as well as in the perivascular space, may lead to the development of new, local biomarkers of coronary inflammation.

### 1.2 Defining epicardial, pericardial/paracardial and perivascular adipose tissue

Human adipose tissue is distributed anatomically into two main compartments, subcutaneous and visceral, and functionally into three depots, white, brown and ‘beige’ or ‘brite’.^13^ White adipose tissue is the main depot for metabolic energy storage, brown adipose tissue for non-shivering thermogenesis, and ‘beige’ refers to brown-like adipocytes inside white adipose tissue that present the ability to produce heat via uncoupling of mitochondrial respiration.^14^ Epicardial adipose tissue (EAT) is predominantly white adipose tissue located between the myocardium and the visceral layer of the pericardium, and it differs functionally and embryologically from pericardial (or paracardial) fat, which is the part of intrathoracic visceral adipose tissue attached to the outside of the parietal layer of the pericardium.^15^ EAT volume measurements are considered a surrogate of metabolically unhealthy obesity and have been used for cardiovascular risk stratification.^16^ Its measurements and clinical implications are described in detail below.

Recent data suggests that the part of EAT in close proximity to the coronary arteries, has very different morphological and functional characteristics compared to the rest of this depot,^17^ creating an unmet need for a clear distinction of perivascular adipose tissue (PVAT) from total EAT. Indeed, PVAT is vaguely defined in most of the literature simply as adipose tissue surrounding the blood vessels. However, from an anatomic point of view, it is also embedded in the vascular wall to a different degree, depending on vessel size. In large vessels PVAT is contiguous with the adventitial layer, whereas in small vessels and microvessels PVAT adipocytes are an integral part of the vascular wall itself.^18^ Given its proximity to or inclusion in the vascular wall, PVAT interacts directly with it in a bidirectional way.

In this review article, we first discuss the biology of PVAT and its contribution to cardiovascular disease, with special focus on its bidirectional communication with the vascular wall. Next, we elaborate on imaging of perivascular fat as a window to vascular inflammation and a promising marker of cardiovascular disease with diagnostic and prognostic value.

## 2 The interplay between adipose tissue and the vascular wall

### 2.1 Effects of adipose tissue on the vascular wall (outside to inside signaling)

**Fig. 1:**
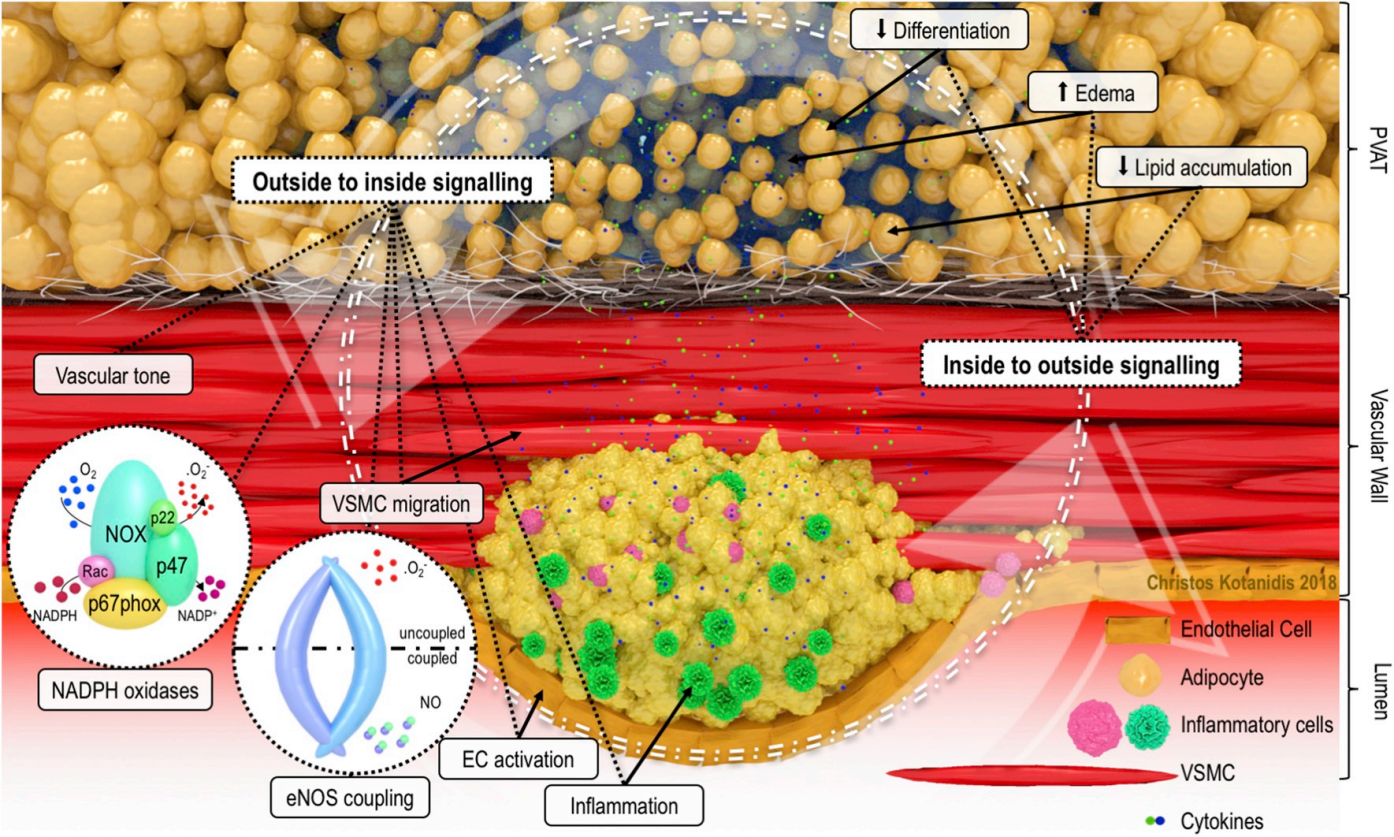
**Cross-talk between the vessel and perivascular adipose tissue.** Perivascular adipose tissue (PVAT) is involved in a bidirectional interplay with the vascular wall. Outside to inside signals, including adipokines (i.e., adiponectin), cytokines, gaseous messengers (such as hydrogen sulfide and nitric oxide), fatty acids, reactive oxygen species, microRNAs and other molecules, are implicated in the regulation of vascular tone, vascular smooth muscle cell (VSMC) migration, regional oxidative stress (NADPH oxidase (NOX) activity and endothelial nitric oxide synthase (eNOS) coupling), inflammation (M1–M2 macrophage polarization), and local endothelial cell (EC) activation. Inside to outside signals, including components of vascular inflammation (such as pro-inflammatory cytokines) and oxidative stress (such as 4-hydroxynonenal), can in turn affect PVAT biology, and more specifically, lead to increased edema and decreased adipocyte size, differentiation and lipid accumulation.

Adipose tissue produces a wide variety of bioactive molecules, ranging from adipokines (such as adiponectin, leptin, apelin) to inflammatory cytokines, micro-RNAs, microvesicles, inorganic molecules such as hydrogen sulfide, reactive oxygen species, fatty acid metabolites and others (Fig. 1). Products from large depots, such as the subcutaneous or visceral adipose tissue, are released into the bloodstream through the adipose tissue microvessels, and act on the cardiovascular system in an endocrine manner.^13^ Products from PVAT can diffuse directly into the vascular wall in a paracrine way, thus directly affecting its biology.^19^ Regardless of the route they employ, adipose tissue-derived products influence many aspects of vascular biology, including vascular tone, inflammation, vascular smooth muscle cell migration, endothelial function and vascular redox state.^12^^,^^20^ We have shown that the secretome of PVAT contains anti-inflammatory and antioxidant substances like adiponectin, and it may host defense mechanisms for the vascular wall.^21^^,^^22^ PVAT also contains inflammatory components. Pro-oxidant adipokines such as leptin and resistin, lead to increased vascular oxidative stress, which is a key contributor of vascular inflammation and has therefore been associated with the process of atherogenesis and adverse clinical outcomes.^22^ Vascular inflammation can be promoted further by expression of endothelial cell adhesion molecules, which is induced by adipokines such as visfatin and adipose tissue-derived pro-inflammatory cytokines such as IL-1β and tumor necrosis factor (TNF).^13^ eNOS coupling status may also be modulated by adipose tissue, leading either to the beneficiary production of NO, or to the damaging production of superoxide, through reduced bioavailability of the enzyme's cofactor tetrahydrobiopterin (BH4).^21^ Moreover, the population of adventitia with migrating inflammatory cells that deposit collagen and lead to localized upregulation of inflammatory chemokines, TGF-β dependent differentiation of fibroblasts to migratory myofibroblasts and increased vasa vasorum neovascularization, have been shown to contribute to atherosclerosis development.^23^ Finally, the immunologic state of adipose tissue, mainly macrophage infiltration and polarization, is described as a crucial regulator of its function, as PVAT inflammation, through the release of a range of cytokines and chemokines, is associated with vascular dysfunction.^24^ It is therefore clear that the vascular wall and its PVAT have a special relationship; understanding how the two organs communicate with each other is critical for the discovery of new therapeutic targets in cardiovascular pharmacology and new biomarkers in cardiovascular diagnostics.

### 2.2 How adipose tissue receives signals from the circulation

Many chronic or end-stage diseases, such as cancer, congestive heart failure, infectious (tuberculosis) and inflammatory diseases (rheumatoid arthritis, Crohn's disease), as well simply being very elderly without obvious disease, can be associated with cachexia.^25^ Cachexia describes a state of chronic low grade inflammation, characterized by a hypercatabolic state leading to unintentional weight loss though lipolysis of adipose tissue.^26^ Systemic low-grade inflammation suppresses adipose-tissue produced adiponectin levels and abolishes its insulin-sensitizing and anti-atherogenic properties, in humans with or without significant cardiovascular disease.^27^ Overall, it is apparent that adipose tissue is a dynamic organ, which undergoes changes, ranging from expansion in the presence of high calorie intake in obesity, to atrophy seen in many conditions presenting with cachexia, to transcriptome changes driven by circulating inflammatory molecules. This understanding of fat, offers useful insight into the so-called obesity paradox, suggesting a ‘U-shaped’ association between Body Mass Index (BMI) and mortality.^28^ The obesity paradox can easily be explained if we accept that while excess adipose tissue is associated with reduced survival, significantly reduced adipose tissue in chronic diseases (e.g. cancer, heart failure, renal failure, chronic inflammatory diseases and other conditions) is also associated with reduced survival rates. Lack of consideration of the negative effects of reduced adipose tissue underlies the misleading concept that there is only a positive association between BMI and short-term (5–10 years) survival in the general population.^29^^,^^30^

### 2.3 PVAT as a sensor of early vascular disease signals (inside to outside signaling)

Adipose tissue is made up of lipid containing cells (adipocytes), stromal cells (preadipocytes, inflammatory cells, fibroblasts and others) and interstitial tissue. The concept that the vascular wall may release signals able to drive changes in the surrounding PVAT has been introduced in an attempt to understand why human arteries with increased levels of oxidative stress have increased release of the antioxidant adipokine adiponectin from their PVAT.^12^^,^^31^ It was then found that products of lipid peroxidation are released from the vascular wall and diffuse into the perivascular space (inside-to-outside signals); The adipocytes within PVAT sense these signals (adipose tissue “sensing”), and by modifying their PPARγ signaling, they increase the release of adiponectin, which is then secreted to exert vasoprotective effects on the adjacent artery (outside-to-inside signal). This proof of principle was then confirmed by other studies showing that vascular injury in mice induces rapid phenotypic changes in PVAT, primarily driven by TNF-α.^32^

**Fig. 2:**
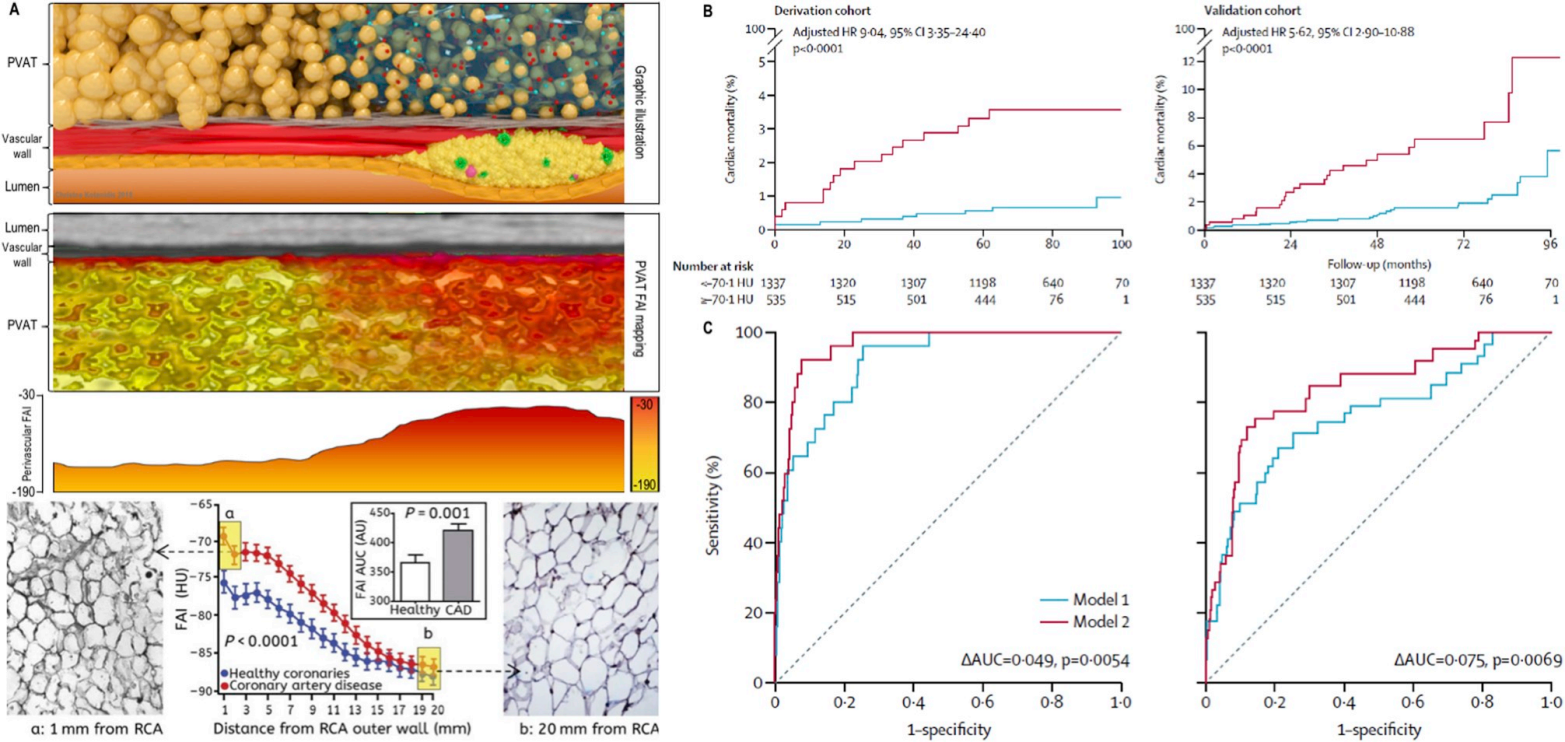
**Perivascular FAI gradient and prognostic value. (A)** Illustrative visualization of the perivascular FAI gradient and radial distance from vascular wall in patients with CAD. **(B)** Kaplan-Meier curves of cardiac mortality with high versus low perivascular FAI for the derivation and validation cohorts. HRs are adjusted for risk factors, technical factors, the extent of coronary artery disease and number of high-risk plaque features. **(C)** Comparison of time-dependent ROC curves (at 6 years) and respective AUC of two nested models for discrimination of cardiac mortality in the derivation (top) and validation (bottom) cohorts. Model 1 represents the current state-of-the-art in risk assessment and consists of age, sex, risk factors (hypertension, hypercholesterolemia, diabetes mellitus, smoker status, epicardial adipose tissue volume), modified Duke coronary artery disease index, and number of high-risk plaque features on coronary CTA. Model 2 incorporates perivascular FAI values into model 1. AUC = area under the curve; FAI = fat attenuation index; PVAT=Perivascular adipose tissue; HR = hazard ratio; HU=Hounsfield unit. Reproduced with permission from Antonopoulos et al.^17^ and Oikonomou et al.^58^

We have recently shown that the inflamed human vascular wall also releases inflammatory cytokines like TNFα, IL-6 and IFNγ, which also diffuse into the perivascular space, triggering a local “cachexia-type” response by the adipocytes within PVAT.^17^ These adipocytes get smaller as their intracellular lipid content depletes due to lipolysis as well as adipogenesis suppression in the microenvironment of the perivascular space (Fig. 2A).^17^ As a result, the inflamed human coronary arteries create a gradient of adipocyte size around them, identified at a macroscopic level by a gradual shift in the balance between the lipid and aqueous phase in the adipose tissue around the inflamed artery.^17^ Indeed, as the adipocytes get smaller in size by reducing their intracellular lipid content, the lipid phase of the tissue is reduced and the aqueous phase increased (due to changes of the adipocyte content but also due to the increased interstitial space). In addition, perivascular edema may also appear as a result of inflammation-induced permeability of the microcirculation around the inflamed artery.

These changes are dynamic, tracking shifts in coronary inflammation in either direction, although the kinetics of these changes are not entirely understood. Following our original observation,^17^ another study confirmed the principle that inflamed arteries drive inflammatory responses in PVAT, by demonstrating that angioplasty-induced coronary injury in a porcine model, leads to a remarkable local uptake of ^18^F-fluorodeoxyglucose (^18^F-FDG) in PVAT, detected by positron emission tomography (PET).^33^ These findings support the basic principle that an “inside to outside” signaling network allows vascular inflammation to be communicated to PVAT, which in turn adjusts its biology by entering a state of hypercatabolism and suppressed adipogenesis (Fig. 1). The possibility to detect these molecular changes around the inflamed human artery using non-invasive imaging would allow us to use them as a “thermometer”, to detect or even quantify coronary inflammation.

## 3 Imaging perivascular and epicardial adipose tissue

Given the limited knowledge of PVAT's biology, imaging of this adipose tissue depot has been confused with imaging of epicardial adipose tissue (EAT). However, the recent scientific discoveries make it clear that PVAT has different biological characteristics than the non-PVAT EAT. Indeed, the composition and distribution of PVAT around the coronary arteries is driven by signals arising from the vascular wall, while the biology of EAT is mainly driven by systemic conditions, like obesity.^17^ Indeed, the volume of EAT has been associated in the past with coronary calcification,^34^ myocardial ischemia^35^ and the severity of coronary atherosclerosis,^36^ confirming that EAT is part of an obesity phenotype influenced by multiple risk factors such as hypertension, hyperlipidemia and diabetes, all part of metabolically unhealthy obesity.

### 3.1 Measuring EAT volume

As noted above, the volume of EAT can be measured by multiple modalities. A description of the function, advantages and drawbacks of these modalities is presented in Table 1. EAT thickness can be measured two dimensionally by standard transthoracic ultrasound as the echo-free space between the outer wall of the myocardium and the visceral layer of pericardium, as proposed by Iacobellis et al.^37^ providing an estimate of EAT volume. This measure has been associated with all features of the metabolic syndrome.^38^ MRI can volumetrically measure EAT volume^39^, but cardiac MRI is not performed as widely as CT and EAT quantification requires tedious manual tracing, although recently described automation tools may be promising.^40^ CT -contrast or non-contrast- is considered to be the gold standard, due to its spatial resolution and volumetric acquisition. In CT, adipose tissue is detected within the window of −30 to −190 Hounsfield Units (HU). The development of software applications dedicated to automated EAT volume quantification such as QFAT^41^ have revolutionized the way we study this adipose tissue depot.^42^ Furthermore, it is increasingly available clinically given the growing use of non-contrast CT for coronary artery calcium assessment as well as of coronary CT angiography. Over time, this measurement has become increasingly automated, and most recently has been quantified automatically directly from non-contrast CT images using deep learning.^43^ EAT volume has been shown to have multiple associations with CAD.^36^^,^^44^ EAT volume by CT has been associated with the presence of ischemic heart disease and coronary calcification progression^45^ as well as adverse cardiovascular events^46^ and ischemia.^47^^,^^48^ In intermediate risk patients, EAT volume has been positively associated with coronary stenosis, ischemia, and high-risk plaque features.^34^^,^^49^^,^^50^ Nonetheless, in subjects with high cardiovascular risk, the association between EAT volume and obstructive coronary artery disease or coronary calcification is reduced,^51^ in accordance with recent basic science findings supporting that extensive inflammation of the coronary arteries can cause local “cachexia”, reducing the fat content in the perivascular space, and this drives down the overall volume of EAT (which includes PVAT and non-PVAT).

**Table 1:** Comparison of noninvasive adipose tissue imaging modalities.

Imaging Modality	Quantitative measures	Qualitative measures	Advantages	Disadvantages
Transthoracic echocardiography	• Thickness	–	• Low cost • Wide clinical availability • Ease of use	• 2D—not volumentric • High operator dependence • No PVAT assessment
CT	• Thickness • Area • Volume	• Attenuation • FAI_PVAT_	• High spatial resolution • 3D volume • Wide clinical availability • Coronary CT angiography • PVAT characterization	• Radiation (and contrast agent) exposure
MRI	• Thickness • Area • Volume	• Proton density fat fraction	• High spatial resolution • 3D volume possible • No radiation • Proton spectroscopy for functional characterisations (in vitro)	• Long scanning duration • High cost • Low clinical availability • No PVAT assessment
^18^F-FDG PET	• SUV	–	• Functional assessment of inflammatory/metabolic activity	• Low spatial resolution • Variable myocardial suppression • High cost • Radiation exposure • Low clinical availability

CT: Computed tomography; MRI: Magnetic resonance imaging; ^18^F-FDG PET: ^18^F-fluorodeoxyglucose positron emission tomography; SUV: standardized uptake values; 2D: two-dimensional; FAI_PVAT_: Perivascular adipose tissue fat attenuation index.

In the SMART study, Franssens et al. found a negative association between EAT attenuation and age, BMI, waist circumference, visceral abdominal adipose tissue, fasting glucose and insulin resistance.^52^ Similarly, a negative correlation between EAT attenuation and coronary calcification was also observed in low-mid risk individuals,^53^ although in higher risk individuals EAT attenuation was paradoxically linked with the presence of acute myocardial infarction.^54^ These contradictory findings can be attributed to a number of factors, such as small sample size, selection bias and methodological limitations, but the most important confounder is the complex biology driving heterogeneity of the molecular phenotypes (and imaging characteristics) of EAT, which fails to distinguish PVAT from non-PVAT.

### 3.2 Measuring PVAT attenuation

We have recently shown by using a radiotranscriptomic approach, that in the presence of large, lipid-full adipocytes, the attenuation of adipose tissue in CT is in the more negative range (towards −190HU), while in the presence of small adipocytes, with less lipid content, the attenuation shifts to the less negative range (towards −30HU), as there is a shift in the tissue composition from the lipid to the aqueous phase^17^ due to several factors: a) reduced intracellular lipid content, b) increased intracellular aqueous phase, which replaces intracellular lipids after lipolysis c) increased extracellular fluid from the shrinking adipocytes and d) edema in the inflamed environment. We have also shown that in large adipose tissue depots like the subcutaneous, the adipocyte size and CT attenuation is driven by systemic features of metabolically unhealthy obesity (i.e. insulin resistance or inherent adipose tissue inflammation), characterized by increased intracellular lipid accumulation and active adipogenesis (i.e. expression of FABP4, CEBPA, PPARγ, PREF1 and others).^17^

In PVAT around the coronaries, the attenuation is affected by inflammatory signals coming from the vascular wall,^17^ which induce an opposite phenotype (lipolysis, suppressed adipogenesis, neoangiogenesis and high micro-vessel permeability leading to perivascular edema). These biological changes generate gradients of the balance between the lipid/aqueous phases, captured as gradients of the CT attenuation in the perivascular space, which run over and above the systemic signals captured by the attenuation of the non-PVAT depots.

PET imaging is the gold standard in imaging of tissue inflammation in vivo, and evaluation of PVAT inflammation assessed by FDG uptake using positron emission tomography (PET), showed greater standardized uptake value (SUV) in pericoronary fat compared with other fat depots and that it is associated with significant coronary artery stenosis.^55^ However, low spatial resolution, high background noise from the myocardial uptake of FDG, high exposure to radiation, and low clinical availability are limiting factors for PET imaging, restricting its use in low risk populations. Further evidence of the relationship between vascular inflammation and PVAT attenuation has recently been shown.^56^ ^18^F—NaF PET vascular uptake, a marker of the rate of microcalcification, has been shown to be increased in ruptured plaques from patients with acute coronary syndromes (ACS).^57^ Further, in a series of stable patients with high risk coronary plaques on coronary CTA, increased attenuation of PVAT was shown to be associated with focal ^18^F—NaF PET uptake around and in the high-risk plaques.^56^ This is an important proof of principle study, confirming the hypothesis that local coronary inflammation related with vulnerable plaques, can be detected non-invasively by studying perivascular attenuation, providing information very similar to ^18^F—NaF PET-CT, by using standard CCTA.

## 4 Imaging PVAT: how to make sense from complex attenuation maps

Artificial intelligence-based image analysis approaches have been developed, and they provide accurate and reproducible measurement of PVAT attenuation (GB2018/1818049.9, Caristo Diagnostics LTD). In recent translational studies exploring the regional biological variability of EAT, we have suggested that for standardization, coronary PVAT around the epicardial coronary arteries, should be defined as the adipose tissue located within a radial distance from the outer vessel wall equal to the diameter of the adjacent coronary vessel.^17^ This is roughly the distance from the coronary arteries after which EAT cellular composition and biological signature reach a “steady state”. By analyzing the three dimensional changes of attenuation within this PVAT space, we can extract information about the inflammatory burden of the adjacent coronary artery.^17^ This information is captured by using AI-enhanced algorithms such as the Fat Attenuation Index (FAI), which represents a measure of weighted attenuation shifts within PVAT. In addition, we have also developed a proof of principle tool called Volumetric Perivascular Characterization Index (VPCI) to capture the attenuation gradients from the outer surface of the vascular wall (PVAT) to the non-PVAT. These analyses are performed by using the standard adipose tissue window of −190 to −30HU, within an algorithm that involves multiple adjustments and is constantly evolving though artificial intelligence (AI) processes, to capture non-invasively PVAT phenotypic alterations, such as reduction in adipocyte size and lipid accumulation, which have been shown to chaperon coronary inflammation.^17^ Quantifying perivascular FAI and extracting meaningful information from perivascular attenuation maps is a particularly complex process. The FAI algorithm quantifies a weighted measure of attenuation in concentric 1 mm-layers of perivascular tissue around the human arterial wall, capturing the respective perivascular attenuation gradients, reflecting the changes in PVAT biology that occur as a result of vascular inflammation. More specifically, the heart is initially segmented and the vessel wall of interest is defined in a fully-automated way by the CaRi-HEART application, followed by analysis algorithms dependent on the type of analysis requested (standardized proximal segment analysis or plaque-specific). For the standardized segment analysis (*e.g.* for the proximal segments of the main epicardial coronary arteries), the longitudinal length of PVAT is determined by the vessel segment and the analysis takes place in multiple layers of PVAT in the perpendicular dimension. This is essential to capture the gradient of PVAT density around the human artery. The plaque-specific analysis is much more complex, and it involves simultaneous scanning of the PVAT in the longitudinal direction (starting from a segment proximal to the lesion of interest -typically 5 mm proximally- and extending to a segment downstream the lesion), while at the same time analyzing PVAT in the perpendicular dimension. It should be noted that FAI is different to the crude “mean CT attenuation (or radiodensity)” of PVAT, since it has to be appropriately corrected and weighted for parameters related to the background status of the adipocytes (obesity effect and others), anatomical factors specific to the coronary segment under investigation, technical scanning parameters (e.g. tube voltage, the reconstruction algorithms used and others), and other information extracted by the FAI algorithm from the CCTA file data. For example, crude measurement of perivascular attenuation, ignoring the attenuation gradients around inflamed arteries, underestimates coronary inflammation in obese individuals who have large overall adipocytes size driving the attenuation closer to −190HU. Similarly, in lean individuals whose adipocytes are smaller, with lower lipid content, crude attenuation measurement overestimates the inflammatory burden of the adjacent artery as the small adipocytes drive attenuation to higher values, even in the absence of local inflammation in the adjacent artery. Measurement of the absolute attenuation values is also affected by the hardware used, CT scan settings, reconstruction algorithms and many other technical parameters, all of which are taken into account for the calculation of FAI. Importantly, FAI is not affected by arterial calcification or lumen attenuation, thus having an advantage over coronary wall biomarkers, although the information provided is complementary to high risk plaque features.^17^

### 4.1 Measuring FAI around the proximal coronary arteries

Originally the measurement of FAI was limited to the proximal 40 mm segments of the right coronary artery, left anterior descending artery and left circumflex artery.^17^ This was due to a lack of molecular validation of FAI measurements in other coronary segments, given that the amount of PVAT and the biological characteristics vary from segment to segment in the coronary tree, and the absolute values of perivascular attenuation are highly dependent on the segment under examination. However, it soon became clear that standardized measurement of perivascular FAI in the proximal 40 mm segments yields comparable results with measurements in the anatomical segments proposed by the SCCT guidelines and provides a sensitive biomarker describing the background inflammatory burden of the entire coronary tree, which may be abnormal even in the absence of any visible coronary atherosclerotic plaque. This is the low-grade inflammation that precedes atherosclerotic plaque formation, and it could potentially have predictive value for future cardiovascular events, as an internal “thermometer” of the entire coronary arterial tree. Perivascular FAI measured in these standardized proximal coronary segments is significantly higher in patients with coronary artery disease compared to individuals without any atherosclerotic plaques (Fig. 2A), while it is not related with local coronary calcification or overall coronary calcium score (CCS), after adjusting for age, gender, and other cardiovascular risk factors, making it a robust biomarker of low-grade, background coronary inflammation.^17^

### 4.2 Plaque (or segment)-specific measurement of FAI

**Fig. 3:**
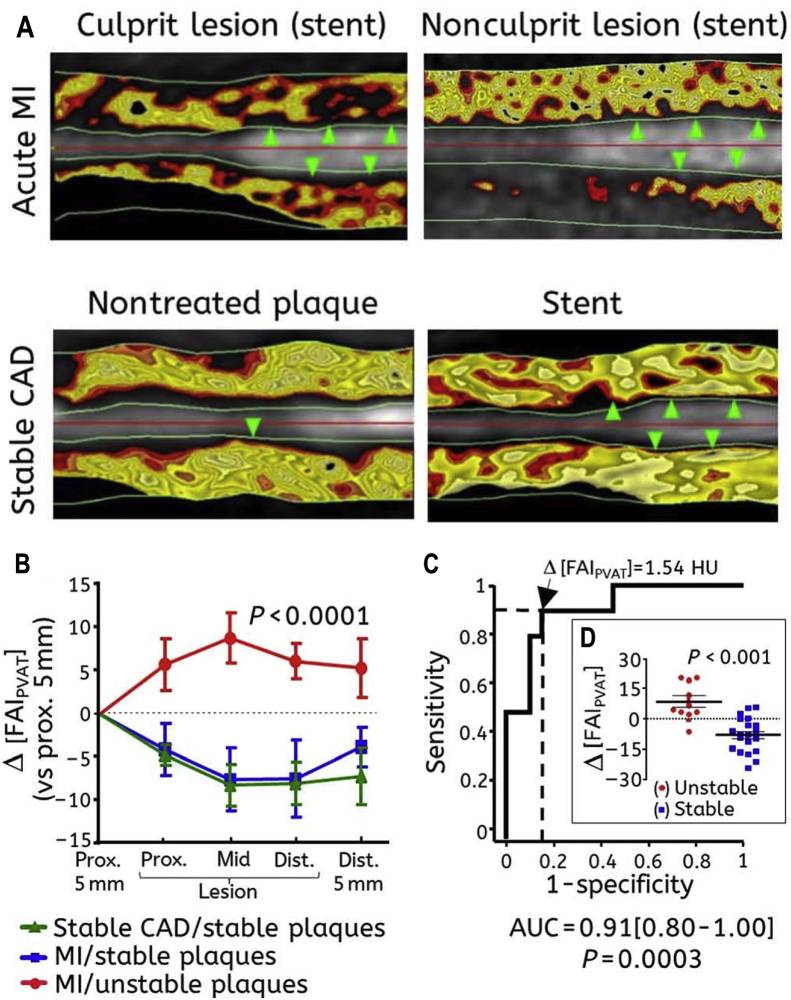
**Perivascular FAI around stable vs non-stable plaques. (A)** Representative images of delineated perivascular fat with FAI colored mapping around: (i) a culprit lesion (green arrows) demonstrating abnormal FAI in a patient with acute MI (upper left image), (ii) a nonculprit lesion (green arrows) from the same patient also showing abnormal FAI (upper right image), (iii) a stable atherosclerotic lesion without a stent (single green arrow, lower left image), and (iv) a stent (green arrows) implanted at least 3 months before imaging (lower right image); black color indicates non-adipose tissue with attenuation values outside the −30 to −190 HU range. **(B)** Perivascular FAI changes around ruptured (culprit) atherosclerotic lesions (n = 10) of patients with acute MI, nonculprit lesions of the same patients (n = 7), or lesions of stable CAD patients (n = 13); **(C and D)** Δ[FAI] comparison between stable and unstable plaques and ROC curve analysis for its diagnostic accuracy in the detection of unstable plaques (culprit lesions); Δ[FAI] = FAI (around lesion) – FAI (proximal segment), FAI = fat attenuation index, CCTA = coronary computed tomography angiography, CAD = coronary artery disease, ROC = receiver operating characteristic, HU=Hounsfield Units. Reproduced with permission from Antonopoulos et al.^17^ (For interpretation of the references to color in this figure legend, the reader is referred to the Web version of this article.)

FAI analysis can be performed around any coronary segment and specifically around atherosclerotic plaques, to test for local variability of coronary inflammation that accompanies the vulnerable plaques. A different type of analysis is required for the use of FAI as a local biomarker of coronary inflammation (i.e. as a relative measure around the plaques under investigation).^17^^,^^58^ Indeed, FAI is significantly increased around the culprit lesions in patients with ACS as compared with non-culprit lesions of the same patient or around stable lesions in stable patients.^17^ Notably, perivascular FAI tracks changes in coronary inflammation following an acute myocardial infarction, as evidenced by a significant decrease of its values around the culprit lesions 5 week after the event^17^ (Fig. 3). To correct for the background effect of the overall inflammation of the coronary tree and enable accurate identification of the local increase of the inflammatory burden of specific “inflamed” plaques, the change of perivascular FAI referenced to a segment proximal to the lesion, was found to be superior to crude perivascular attenuation measurement,^17^ although the magnitude of the changes in PVAT attenuation around culprit lesions during ACS is so high, that measurement of the crude PVAT attenuation can also contribute to the detection of vulnerable plaques.^59^ Importantly, a shift of perivascular attenuation was observed around coronary segments with atherosclerotic plaques compared with segments without disease.^60^ As noted above, increased PVAT attenuation has been associated with increase in ^18^F—NaF PET uptake in stable patients with high risk plaques on CTA,^56^ further confirming the ability of perivascular attenuation shifts to detect coronary inflammation. Other vascular pathologies related with vascular inflammation, such as coronary dissection, are also linked with high attenuation values closer to the vascular wall, confirming the proof of principle, that acute local inflammation in the vascular wall drives shifts of perivascular attenuation.^61^

Further, compared to local perivascular FAI calculation around specific plaques (plaque-specific analysis), determining perivascular FAI around every segment of the coronary tree in a meaningful way, is challenging, due to the anatomical variability of the coronary arteries and the biological variability of local PVAT. This issue has been resolved recently using AI, and a different FAI algorithm is now used for each coronary segment (Caristo Diagnostics, unpublished data). Given the complexity of the perivascular attenuation data interpretation, extracting meaningful information from PVAT attenuation maps requires either interpretation against personalized reference scales (which would make their use non-practical), or incorporation of all corrections in a single AI-enhanced biomarker, like FAI.^17^ Due to the nature of perivascular FAI as a “clever” AI biomarker, the way it is calculated is continuously adjusted as more data from large cohorts become available.

### 4.3 Perivascular FAI and prediction of long-term outcomes

Since inflammation is a key feature of atherogenesis and atherosclerotic plaque rupture, a method determining inflammation in the coronary arteries could have the ability to predict future heart attacks. The CRISP-CT study^58^ explored the ability of perivascular FAI around the proximal segments of the three main epicardial arteries, to predict clinical outcomes in ∼4000 individuals undergoing CCTA as part of their clinical care in Erlangen, Germany and Cleveland, USA (as derivation and validation cohorts respectively, with ∼2000 individuals each). There was a J-shape association between perivascular FAI and the risk for cardiac death. Among individuals with perivascular FAI > -70.1HU, the risk for all-cause mortality was HR (95%CI) 2.55 (1.65–3.92) for the derivation and 3.69 (2.26–6.02) for the validation cohort. Importantly, patients with “abnormally high” perivascular FAI had ∼9 times higher risk for cardiac mortality in the derivation cohort and 5.6 times higher risk of cardiac death (Fig. 2B). This improved prediction of mortality was over and above the current state of the art in risk assessment using CCTA, that includes clinical risk factors, calcium score, the extend of coronary atherosclerosis and the presence of high-risk plaque features (with a delta AUC 0.049 [p = 0.0054] in the derivation and 0.075 [p = 0.0069] in the validation cohorts) (Fig. 2C). In addition, abnormal perivascular FAI was also predictive of non-fatal heart attacks, but was only weakly correlated with calcium score or plasma hsCRP (marker of systemic inflammation). This permits reclassification of an individual's risk, independently of their background calcium score, the presence of CAD or any high risk plaque features.^58^ Interestingly, perivascular FAI lost its predictive value among those individuals who started treatment with statin and aspirin immediately after CCTA, while among those who didn't change their medication, the hazard ratio for cardiac mortality was more than doubled. This suggests that the risk identified by perivascular FAI is modifiable and could potentially be tracked by repeat CCTA after treatments initiation. With respect to the predictive value of FAI in secondary prevention, it has to be noted that the vast majority of the participants in the CRISP-CT study were low-intermediate risk individuals, which are commonly the ones referred for CCTA. Regarding higher risk populations, although the numbers of patients with CAD in the CRISP-CT study were rather low (467 in the derivation and 286 in the validation cohort), the HR for fatal heart attacks remained significant (8.54[2.41-30.21] and 3.85[1.21-12.27] in the derivation and validation cohorts, respectively). The predictive value also remained significant in those with or without high-risk plaque features.

### 4.4 Perivascular FAI: future perspectives and limitations

Thus far, the relation of perivascular FAI with future outcomes has been well described and established, allowing for a unique risk classification system with strong implications for guiding medical management in patients undergoing CCTA. Incorporating perivascular FAI in standard CCTA reporting could guide the use of primary and secondary prevention measures. While the extent of coronary atherosclerosis, stenosis, high risk plaque features, noncalcified plaque measurements, and functional assessments such as FFRct provide powerful risk stratification of patients with CAD, these anatomic assessments, however, do not provide information regarding the activity of CAD. By assessing vascular inflammation, abnormal perivascular FAI, may provide guidance for expensive, novel risk-reducing therapeutics (e.g. anti-PCSK9 inhibitors or Canakinumab). Further, in patients with high-risk anatomic lesions, FAI findings could modify the clinical decisions regarding coronary revascularization in stable ischemic heart disease. In those with normal FAI, aggressive medical management may be appropriate, reserving the invasive procedures for patients defined by FAI as having active disease. While ^18^F—NaF PET uptake could provide similar guidance, it is a separate procedure with substantial additional costs, unlike the FAI which is performed on any CCTA without additional testing. Nevertheless FAI analysis can be challenging given the complexity of calculations involved, and its implementation postulates the need for dedicated powerful workstations. Moreover, there is a need for further validation of the FAI biomarker in specific populations under-represented in CRISP-CT, such as patients with kidney disease. Finally, ongoing clinical trials using coronary FAI are underway to document further the dynamic nature of perivascular FAI in response to treatments, which would make it an attractive solution as a biomarker to test responsiveness to routine or novel therapeutics. In this way, perivascular FAI could be used as a “companion diagnostic”, to CCTA, contributing to the deployment of personalized therapeutic solutions in primary and secondary prevention.

## Conflicts of interest

CA is a founder, shareholder and director of Caristo Diagnostics, a spinout company of the University of Oxford. CA is also director of Oxford Academic Cardiovascular CT Core lab.
